# Genotypic spectrum of α-thalassemia and β-thalassemia in newborns of the Li minority in Hainan province, China

**DOI:** 10.3389/fped.2023.1139387

**Published:** 2023-03-20

**Authors:** Kun Zhong, Haijie Shi, Wenli Wu, Haizhu Xu, Hui Wang, Zhendong Zhao

**Affiliations:** ^1^ National Center for Clinical Laboratories, Institute of Geriatric Medicine, Chinese Academy of Medical Sciences, Beijing Hospital/National Center of Gerontology, Beijing, China; ^2^ Department of Gastroenterology, Hainan General Hospital, Hainan Affiliated Hospital of Hainan Medical University, Haikou, China; ^3^ Hainan Women and Children's Medical Center, Haikou, China; ^4^ Department of Maternal and Child Health, School of Public Health, Peking University, Beijing, China

**Keywords:** newborns, thalassemia, genotypes, Li minority, Hainan provice

## Abstract

**Purpose:**

To explore the genotypes and allele frequencies of α, β and α+β thalassemias in Li minorities, which resided in Hainan Province of China for a long time.

**Methods:**

In the present study, 1,438 newborns of the Li minority were collected from January 2020 to April 2021. The genotypes of thalassemia were detected by fluorescence PCR and verified by flow-through hybridization PCR analyses. Rare genotypes were detected by restriction fragment length polymorphism electrophoresis and Sanger DNA sequencing.

**Results:**

Among 1,438 participants, 1,024 (71.2%) were diagnosed with any kind of thalassemia. Among all thalassemia carriers, 902 (88.09%) subjects were diagnosed with α-thalassemia, and 18 subtypes of α-thalassemia were detected, with the top three genotypes being −α^4.2^/αα (25.39%), −α^3.7^/αα (22.62%) and α^WS^α/αα (16.96%). Thirty-two (3.13%) patients were β-thalassemia carriers, and 6 types of β-thalassemia genotypes were detected. The top two genotypes were β^CD41–42^/β^N^ (46.88%) and β^−28^/β^N^ (18.75%). Additionally, 90 (8.79%) cases were α + β-thalassemia, and the top two genotypes were −α^3.7^/αα, β^CD41–42^/β^N^ (30.00%) and −α^4.2^/αα, β^CD41–42^/β^N^ (26.67%). Furthermore, two genotypes (−α^4.2^/HKαα and β^CD76 GCT > CCT^/β^N^) were first identified in Hainan Province_,_ and β^CD76 GCT > CCT^/β^N^ was first identified in China.

**Conclusion:**

Newborns of Li have a higher prevalence of thalassemia for a long period, and further education on the impact of thalassemia, follow-up studies of the clinical manifestation and treatment and proper intervention methods should be designed to reduce the burden of thalassemia and enhance the quality of life in Li newborns.

## 1. Introduction

Thalassemias are a group of inherited monogenetic blood disorders (α, β, γ, δ, δβ, and εγδβ) that are distributed worldwide. There are two main types of thalassemias, alpha thalassemia (α-thalassemia) and beta thalassemia (β-thalassemia) (1). Thalassemia α and β are particularly spread in regions from the Mediterranean basin to the Middle East, Indian subcontinent, Southeast Asia, Melanesia, and the Pacific Islands (2–4). The severity of thalassemias can vary from mild anemia to stillbirth, which is dependent on the impaired number of corresponding hemoglobin chains (α and/or β). The imbalanced α/β-globin chain ratio was attributed to ineffective erythropoiesis and finally resulted in anemia (5, 6). Previous studies have suggested that thalassemia could be screened and prenatally diagnosed, and it was the most cost-effective method to reduce the occurrence of severe symptoms among newborns (7, 8). Recently, a meta-analysis indicated that the prevalence of thalassemia in China is mainly distributed in southern China, especially in Guangxi (19.10%), Guangdong (11.9%) and Hainan (12.95%) provinces (9, 10). Those three cities had a high prevalence above 10% in China and a more specific prevalence of α-thalassemia (11).

Ethic Li is a Chinese minority and mainly lives in Hainan Province. They were considered the earliest residents on Hainan Island. The population size of the Li minority reached 1.6 million in 2020 (12). The tradition and economic status of the Li people remain similar due to their concentrated residence. Certain studies have shown that the Li people have a higher prevalence of thalassemia than other minorities (13–15). However, most studies have been carried out in Li's adults, in whom the genotypes might be different from those of newborns due to survivor bias, which hampers precise and immediate treatment marking and proper prevention policy design.

Therefore, in the present study, we describe the frequencies of α- and β-thalassemia and specific genotypic mutation frequencies in newborns of the Li minority in Hainan Province.

## 2. Materials and methods

### 2.1. Subjects

From January 2020 to April 2021, we collected 1,517 live births of Li in the Hainan Li Autonomous Region. Among all lived births, approximately ninety-five percent of newborns' parents signed the informed consent form and volunteered to participate in thalassemia genotype testing. The age of newborns ranged from 3 to 20 days. The numbers of boys and girls were 743 and 695, respectively. This study was approved by the Ethics Committee for Clinical Investigation of Hainan Women and Children's Medical Center in 2021 (No. 028). Written informed consent to participate in this study was provided by the participants' legal guardian/next of kin. No potentially identifiable human images or data are presented in this study.

### 2.2. DNA extraction

According to the People's Republic of China Health Industry Standard, “Basic dataset of children ‘s health—Part 3: Newborn screening” (WS 376.3-2013) (16), the trained health worker of maternal and child health care centers in Hainan Li Autonomous Region collected the heel blood of newborns between 3 and 20 days after delivery. Wiping off the first drop of heel blood, the following drops were dripped vertically on 903-filter paper to generate a spot with a diameter over 8 mm. All dried blood spots were sent to the molecular biology laboratory of Hainan Women and Children's Medical Center for genetic analyses with cold-chain transportation. At least six dried blood spots 3 mm in diameter were punched (Panthera-Puncher^TM^ 9 puncher (Perkin Elmer Co., Ltd, Shanghai, Finland)) and placed in a centrifuge tube for DNA extraction. In the screening stage, DNA extraction was performed with the magnetic bead method (Zeesan Biotech Co., Ltd, Xiamen, China) with a Lab-Aid 824s Nucleic Extraction System (Zeesan Biotech Co., Ltd, Xiamen, China). For the verification stage, genomic DNA was extracted with the spin column method (Hybribio Co., Ltd, Guangzhou, China).

### 2.3. Screening and verification of thalassemia genotypes

In the screening stage: the fluorescence Polymerase Chain Reaction -melting curve analyses (SLAN-96S Automatic Medical PCR Analysis System, Shanghai Hongshi Medical Technology Co., Ltd, Shanghai, China) was used to detect the genotype of thalassemia including three α-thalassemia gene deletions (−α^3.7^, −α^4.2^, –^SEA^), and three nondeletion α-thalassemia mutations: Hb CS, Hb QS and Hb Westmead and 21 common β-thalassemia mutations [the names given by HGVS are in parentheses following the names of base changes: −90 (C > T) (c.-140C > T), −73 (A > T) (c.-123A > T), −28 (A > G) (c.-78A > G), −29 (A > G) (c.-79A > G), −30 (T > C) (c.-80T > C), −31 (A > C) (c.-81A > C), −32 (C > A) (c.-82C > A), CD14-15 (+G) (c.45_46insG), CD15-16 (+G) (c.48_49insG), CD17 (A > T) (c.52A > T), CD26 (G > A) (c.79G > A), CD30 (A > G) (c.91A > G), IVS-I-1 (G > T) (c.92 + 1G > T), IVS-I-5 (G > C) (c.92 + 5G > C), CD27-28 (+C) (c.84_85insC), CD37 (G > A) (c.113G > A), CD71-72 (+A) (c.216_217insA), CD43 (G > T) (c.130G > T), CD41-42 (-TTCT) (c.124_127delTTCT), IVS-II-5 (G > C) (c.315 + 5G > C) and IVS-II-654 (C > T) (c.316-197C > T)]. The α-thalassemia, β-thalassemia, nondeletion and α+β thalassemia were detected with the thalassemia genotype detection kit (Zeesan Biotech Co., Ltd, Xiamen, China). The polymerase chain reaction (PCR) was carried out with the SLAN-96S Automatic Medical PCR Analysis System (Shanghai Hongshi Medical Technology Co., Ltd, Shanghai, China).

To ensure the accuracy of the genotyping, certain percentage samples were verified with flow-through hybridization analysis (HB-2012A nucleic acid hybridization, Hybribio Co., Ltd, Guangzhou, China). The genotype kit for thalassemia detection (Hybribio Co., Ltd, Guangzhou, China) included three common α-globin gene deletions, 3 nondeletion α-thalassemia mutations and 19 common β-thalassemia mutations. Moreover, the mutation of −α4.2/HKαα was detected by restriction fragment length polymorphism (RFLP) electrophoresis (Hybribio Co., Ltd, Guangzhou, China) and the mutation of β ^CD76^ GCT^ ^>^ ^CCT(NM_000518.5 (HBB):c.229G^ ^>^ ^C)/β^N^ was detected by Sanger DNA sequencing (Zeesan Biotech Co., Ltd, Xiamen, China).

### 2.4. Statistical analysis

Data and results were analyzed using SPSS version 13.0 (IBM Inc., Chicago, United States). To compare the thalassemia gene-carrying rates among sexes and subgroups, the chi-square (*χ^2^*) test was used. Statistical significance was set at *p *< 0.05 for two tails.

## 3. Results

### 3.1. The prevalence of thalassemia between girls and boys

The numbers of boys' and girls' carriers were 524 and 500, respectively. Boy and girl thalassemia gene-carrying rates were 70.5% (524/743) and 71.9% (500/695), respectively. There was no statistically significant difference between the gene-carrying rates of thalassemia between boys and girls (*χ^2 ^*= 0.074, *P *= 0.786, Table 1).

**Table 1:** The numbers of newborns and gene-carrying rates of thalassemia of newborns in Li people from January 2020 to April 2021 in the Hainan Li autonomous region.

Sex	No. of newborns	No. of newborns tested	Testing rates (%)	No. of newborns carrying gene of thalassemia	Gene-carrying rates of thalassemia (%)
Boy	752	743	98.8	524	70.5
Girl	765	695	90.8	500	71.9
Total	1,517	1,438	94.8	1,024	71.2

### 3.2. The genotypes of α-thalassemia and β-thalassemia

Among 1,438 newborns of Li minorities, 1,024 (71.21%, 1,024/1,438) thalassemia carriers were detected. As shown in Table 2, 902 (88.09%, 902/1,024) cases with α-thalassemia and 18 different types of α-thalassemia were detected. The top three genotypes were −α^4.2^/αα (25.39%, 229/902), −α^3.7^/αα (22.62%, 204/902) and α^WS^α/αα (153/902, 16.96%). For β-thalassemia, 32 (3.14%, 32/1,024) were cases, and six different types of genotypes were detected (Table 3). The top two genotypes were β^CD41–42^/β^N^ (46.88%, 15/32) and β^−28^/β^N^ (18.75%, 6/32).

**Table 2:** Distribution of α-thalassemia genotypes in the newborns of Li in Hainan province.

α-thalassemia genotype	Type	Cases	Frequency (%)
−α^4.2^/αα	α^+^/α	229	25.39
−α^3.7^/αα	α^+^/α	204	22.62
α^WS^α/αα	α^+^/α	153	16.96
−α^4.2^/α^WS^α	α^+^/α^+^	63	6.98
−α^3.7^/−α^4.2^	α^+^/α^+^	61	6.76
−α^3.7^/α^WS^α	α^+^/α^+^	51	5.65
−α^3.7^/−α^3.7^	α^+^/α^+^	35	3.88
−α^4.2^/−α^4.2^	α^+^/α^+^	32	3.55
α^QS^α/αα	α^+^/α	27	2.99
−α^3.7^/α^QS^α	α^+^/α^+^	14	1.55
–^SEA^/−α^3.7^	α^0^/α^+^	8	0.89
–^SEA^/−α^4.2^	α^0^/α^+^	6	0.67
–^SEA^/αα	α^0^/α	6	0.67
–^SEA^/α^WS^α	α^0^/α^+^	4	0.44
−α^4.2^/α^QS^α	α^+^/α^+^	4	0.44
−α^3.7^/α^CS^α	α^+^/α^+^	2	0.22
α^QS^α/α^WS^α	α^+^/α^+^	2	0.22
−α^4.2^/HKαα	α^+^/α^+^	1	0.11
Total		902	100

**Table 3:** Distribution of β-thalassemia genotypes in the newborns of Li in Hainan province.

Genotypes	HGVS name	Type	Cases	Frequency (%)
β^CD41–42^/β^N^	HBB:c.126_129delCTTT	β^0^/β	15	46.88
β^−28^/β^N^	HBB:c.-78A > G	β^+^/β	6	18.75
β^IVS−II−654^/β^N^	HBB:c.316-197C > T	β^+^/β	5	15.63
β^CD17^/β^N^	HBB:c.52A > T	β^0^/β	3	9.38
β^IVS−I−1^/β^N^	HBB:c.92 + 1G > T	β^0^/β	2	6.25
β^CD76 GCT > CCT^/β^N^	NM_000518.5 (HBB):c.229G > C (*p*.Ala77Pro)	β^+^/β	1	3.13
Total			32	100

HGVS, human genome variation society; β^N^, N means normal beta chain.

### 3.3. The genotypes of α+β-thalassemia

As shown in Table 4, 90 (8.79%, 90/1,024) cases with composite α+β-thalassemia were detected. The two common genotypes were −α^3.7^/αα+β^CD41–42^/β^N^ (30.00%, 27/90) and −α^4.2^/αα+β^CD41–42^/β^N^ (26.67%, 24/90). Two rare genotypes, −α^4.2^/HKαα and β^CD76 GCT > CCT^/β^N^ [NM_000518.5 (HBB):c.229G > C (*p*.Ala77Pro)]), were detected. The mutation of −α^4.2^/HKαα was detected for the first time in Hainan Province. The mutation β^CD76 GCT > CCT^/β^N^ was detected for the first time in China.

**Table 4:** Distribution of composite α + β thalassemia in the newborns of Li in Hainan province.

Genotypes	Type	Cases	Frequency (%)
−α^3.7^/αα, β^CD41–42^/β^N^	α^+^/α, β^+^/β^0^	27	30.00
−α^4.2^/αα, β^CD41–42^/β^N^	α^+^/α, β^+^/β^0^	24	26.67
−α^3.7^/−α^4.2^, β^CD41–42^/β^N^	α^+^/α^+^, β^+^/β^0^	14	15.56
−α^3.7^/α^WS^α, β^CD41–42^/β^N^	α^+^/α^T^, β^+^/β^0^	10	11.11
−α^3.7^/−α^3.7^, β^CD41–42^/β^N^	α^+^/α^+^, β^+^/β^0^	8	8.89
−α^4.2^/α^WS^α, β^CD41–42^/β^N^	α^+^/α^T^, β^+^/β^0^	3	3.33
α^WS^α/αα, β^CD41–42^/β^N^	α^T^/α, β^+^/β^0^	1	1.11
−α^4.2^/αα, β^IVS−II−654^/β^N^	α^+^/α, β^+^/β^0^	1	1.11
Total		90	100.00

### 3.4. The allele frequency of α- and β-thalassemia mutations

As shown in Table 5, 1,312 chromosomes carrying α-thalassemia gene mutations were identified, including seven α genetic mutations. The top three most frequent mutations were −α^3.7^ (36.36%, 477/1,312), −α^4.2^ (35.82%, 470/1,312) and α^WS^α (21.88%, 287/1,312). As shown in Table 6, 120 chromosomes carrying β-thalassemia gene mutations were identified, including six β gene mutations. The top three most frequent mutations were CD41-42 (85.00%, 102/120), CD41-28 (5.00%, 6/120) and IVS-II-654 (5.00%, 6/120).

**Table 5:** Allele frequencies of α-thalassemia mutations in the newborns of Li in Hainan province.

Affected alleles	HGVS name	Number of Allele	Frequency (%)
−α^3.7^	NG_000006.1:g.34247_38050del	477	36.36
−α^4.2^	NC_000016.10:g.169818_174075del	470	35.82
α^WS^α	HBA2:c.369C > G	287	21.88
α^QS^α	HBA2:c.377T > C	49	3.73
–^SEA^	NC_000016.10:g.165401_184701del	24	1.83
α^CS^α	HBA2:c.427T > C	4	0.30
HKαα	N/A	1	0.08
Total		1,312	100.00

HGVS, human genome variation society.

**Table 6:** Allele frequencies of β-thalassemia mutations in the newborns of Li in Hainan province.

Affected allele	Number of affected alleles	Frequency (%)
β^CD41–42^	102	85.00
β^−28^	6	5.00
β^IVS−II−654^	6	5.00
β^CD17^	3	2.50
β^IVS−I−1^	2	1.67
β^CD76 GCT > CCT^	1	0.83
Total	120	100.00

## 4. Discussion

In the present study, we focused on the newborns of the Li minority who have abundant genotypes of thalassemia and are living in the high prevalence malaria region (Li Autonomous Region of Hainan) in China. The rank of thalassemia types was α-thalassemia (88.09%, 902/1,024), β-thalassemia (3.14% (32/1,024) and α + β (8.79%, 90/1,024). Additionally, we first found the HKαα allele and β^CD76 GCT > CCT^/β^N^ allele in the Li minority.

The data of the present study are in line with those of previous studies, and α-thalassemia is the major type of thalassemia in the Li minority (14, 15). The proportions of different subtypes of α thalassemia and β-thalassemia are mainly consistent with one recent study carried out in childbearing age adults of the Li minority (15), particularly for the top two genotypes in α thalassemia (−α^4.2^/αα and −α^3.7^/αα) and top three in β-thalassemia (β^CD41–42^/β^N^, β^−28^/β^N^ and β^IVS−II−654^/β^N^). However, the percentages in different subtypes had significant disparities; for example, β^CD41–42^/β^N^ only accounted for 46.88% in our present study, but it accounted for 90.96% in childbearing age adults of the Li minority (15). A similar scenario was also observed in α thalassemia; −α^4.2^/αα accounted for 25.39% and −α^3.7^/αα accounted for 22.62%, but those two subtypes accounted for 17.24% and 17.16%, respectively, in childbearing age adults in the Li minority (15). The plausible reason might be that our sample was only collected from Li minority autonomous regions, and Wang's research was conducted with Li minorities from 19 cities and counties of Hainan. With the development of economic and transportation technology, increasingly more Li travelled around Hainan Province. Interestingly, another recent study carried out with 1to10-year-old children in Guangxi by He revealed that–-SEA and–-SEA/αα were the most frequent genotypes in α thalassemia. Nonetheless, the major type of β-thalassemia is β^CD41–42^/β^N^ (17). Meanwhile, those major types of thalassemia were consistent with the residents living in the Dongguan region of Guangdong Province (18). However, it seems that the southern part of China has different major genotypes of thalassemia in comparison with Chongqing/Yunnan province/Zhejiang province/northern part of China, where β-thalassemia is the dominant genotype (19–22). These results proved that there were regional differences in thalassemia (23). The possible reason might be the inbreeding and migration of human beings. From the route out of Africa, we do observe that human beings first settled near the coast (24). Li minority was the aborigines of Hainan Island, who had been living on Hainan Island for more than 3,000 years. More genetic immigration tracing could be conducted to gain insight into the history of thalassemia in the Li minority.

Notably, the detection rates of nondeletion α-thalassemia mutations were much higher than those in other regions of China (19–22). The gene mutation frequencies of α^WS^α, α^QS^α and α^CS^α were 21.88%, 3.73% and 0.30%, respectively, which are relatively higher than those in a previous report (13, 25). The reason why the prevalence of nondeletion α-thalassemia was very high might be attributed to two aspects. First, the cases and subjects of many previous reports were collected from health examinations, genetic counselling and prenatal diagnosis, which might omit some carriers with no counselling needs or mild symptoms. Second, we speculated that the traditional culture of the Li minority, consanguineous marriage, was very common in the Li minority, which might be susceptible to inheriting mutations and insufficient attention to the harm of thalassemia due to peer effects.

Two rare genotypes, −α^4.2^/HKαα and β^CD76 GCT > CCT^/β^N^ (c.229G > C), were detected in the present study. −α^4.2^/HKαα was identified for the first time in Hainan Province, and currently, no documents have recorded its impact on erythropoiesis. β^CD76 GCT > CCT^/β^N^ was identified for the first time in China. β^CD76 GCT > CCT^/β^N^ (c.229G > C) is a missense mutation. H Wajcman et al. reported only one case of this mutation worldwide before 1990 (26). They believed that it was a new variant of hemoglobin with a greater reduction in oxygen affinity than others. There was no functional evidence of this mutation in the ClinVar database. According to the guidelines of the American College of Medical Genetics (ACMG), this variant is preliminarily considered a deleterious mutation. Follow-up studies need to be performed to observe and record how it impacts the erythropoiesis of carriers, and appropriate invention methods should be conducted when the carrier needs.

For the newborn population, the traditional screening method for thalassemia, which is a combination of complete blood count (CBC) and hemoglobin electrophoresis, has many disadvantages, such as repeated blood sampling, higher cost and longer waiting time. In contrast, thalassemia genetic testing is performed by testing dried blood spots on filter paper, which does not require fresh blood sampling and can be easily retested. Genetic testing is conducive to population screening and has been clinically verified in some studies (27, 28).

In view of the high carrying rates of thalassemia genes in newborns of the Li minority, applicable and feasible interventions should be designed to reduce the prevalence. In the response to the call of “Thalassemia in children: from quality of care to quality of life”, prenatal and neonatal screening of thalassemia disease is critical to alert public awareness of this disease (29). Previous research indicated that children with thalassemia had significantly lower quality of life in the physical, emotional, social and school functioning domains (30, 31). This situation would be even worse in low-resource settings, for instance, Li minority. When children grow up, their general health is impacted as well, with a smaller impact on mental health (32). Additionally, caregivers simultaneously suffer from anxiety and depression (33). Overall, the disease burden is not only limited to the patients themselves. The urbanization index and health care system of Hainan Province are relatively low in comparison with those of Beijing or Shanghai. Compared with other congenital disorders of newborns, the prevalence of thalassemia was high in the Li minority (34). The Chinese National Center for Clinical Laboratories has been ensuring the quality of newborn screening work over the years (35). However, quality of life care should also catch up and be implemented step by step. For instance, public education of the impact of thalassemia disease could be embedded in the pregnancy school to further enhance the voluntary screening rate. During the pregnancy school, let the pregnant women and their husband understand this disease and know the typical syndrome of this disease and get help from the hospital in the proper time are essential as well. Since the health worker force is limited in Hainan as well, online courses would be another way for pregnant women and unmarried young people as well. Additionally, free counseling and proper portion reimbursement of the costing in transfusion-dependent thalassemia could also be designed to bridge the generation gap by policy.

Our study is the first to comprehensively profile the genotypes of thalassemia in newborns among the Li minority. Second, neonatal genetics were analysed for thalassemia. This would provide additional information about the spectrum of genotypes of thalassemia for the Li minority. Finally, the accuracy of the present study is relatively high due to the application of screening and verification methods.

However, several limitations need to be addressed in the present study as well. Due to data availability, we could not include all Li minorities in Hainan, which might have different frequencies of certain subtypes of thalassemia. Second, we only decipher the genetic variants of thalassemia, and no clinical manifestation was followed-up, which hampers the design of proper and precise interventions in the public.

## 5. Conclusion

The newborns of the Li minority have a high prevalenceof thalassemia genotypes. Screening work should accompany education to enhance the awareness and recognition of symptoms of this disease for health care needs. To further enhance the quality of life among thalassemia carriers, follow-up studies need to be designed.

## Data Availability

The original contributions presented in the study are included in the article/Supplementary Materials, further inquiries can be directed to the corresponding authors.

## Impact statement

This study was the first comprehensive study using neonatal samples and molecular biology methods to investigate the frequency of genotypes among α-, β- and α+β thalassemia in newborns of the Li minority in southern China. Using the newborn to describe the genotypes and mutation frequency could provide additional information about the genotypes of thalassemia for the Li minority. The newborns of Li people had a relatively higher incidence and rare genotypes of thalassemia than Chinese Han people. Appropriate public health interventions should be designed for this population.
